# Sinonasal teratocarcinosarcoma: a case report

**DOI:** 10.1186/s13256-017-1327-y

**Published:** 2017-06-22

**Authors:** Yi Chao Foong, Vince Murdolo, Nusa Naiman, Laura Hepner, Raef Awad

**Affiliations:** 1 Department of Radiation Oncology, Royal Hobart Hospital, 17 Liverpool St, Hobart, Tasmania 7000 Australia; 2 University of Tasmania, Hobart, Tasmania Australia; 3 Department of Pathology, Royal Hobart Hospital, Hobart, Tasmania Australia; 4 Department of Ear, Nose and Throat Surgery, Royal Hobart Hospital, Hobart, Tasmania Australia

**Keywords:** Sinonasal teratocarcinosarcoma, Intensity-modulated radiation therapy, Head and neck malignancy, Ear nose and throat surgery, Histopathology, Case report, Malignancy, Radiotherapy, Radiation oncology

## Abstract

**Background:**

Sinonasal teratocarcinosarcoma is a rare and aggressive malignancy with histological features of both carcinosarcoma and teratoma. The optimal management of this malignancy is unclear, with most patients being managed by a combination of surgery and radiotherapy.

**Case presentation:**

We describe an 83-year-old white woman with sinonasal teratocarcinosarcoma of her left nasal cavity treated with surgical debulking initially with radiological evidence of residual disease which was treated with radiotherapy (60 Gy in 30 fractions). A follow-up examination at 2 years showed no evidence of recurrence.

**Conclusions:**

In cases of sinonasal teratocarcinosarcoma with residual disease post-surgery, radiotherapy alone can be an effective option.

## Background

Sinonasal teratocarcinosarcoma (SNTCS) is an extremely rare malignant neoplasm of uncertain histogenesis [1]. It is highly malignant and locally aggressive, with a high recurrence rate. Almost all cases are found in adults with a significant male predominance [2]. Histopathological findings of this tumor comprise a complex malignant neoplasm that has combined features of teratoma and carcinosarcoma. Phenotypically, these tumors are composed of benign neural elements and various malignant epithelial and mesenchymal components [3]. The clinical features and treatment of patients with these tumors, therefore, remains to be addressed [4]. Here we present a case of SNTCS in an 83-year-old woman treated with debulking surgery and postoperative intensity-modulated radiation therapy (IMRT). There is no evidence of recurrence or complications at 2-year follow-up. This case highlights that radiotherapy alone can be effective post-surgery, and that IMRT is a promising modality in the treatment of SNTCS.

## Case presentation

An 83-year-old white woman presented to her general practitioner initially with 1 month of nasal obstruction, intermittent epistaxis, and anosmia. This is on the background of atrial fibrillation, hypertension, and osteoarthritis. Her regular medications were apixaban, digoxin, amlodipine/valsartan, frusemide, metoprolol, and paracetamol with no known drug allergies. She lived with her son, mobilized independently with a walking stick, and was independent with all activities of daily living (ADLs) with an Eastern Cooperative Oncology Group (ECOG) score of one. She did not smoke tobacco and drank three to four standard units of alcohol per day.

**Fig. 1:**
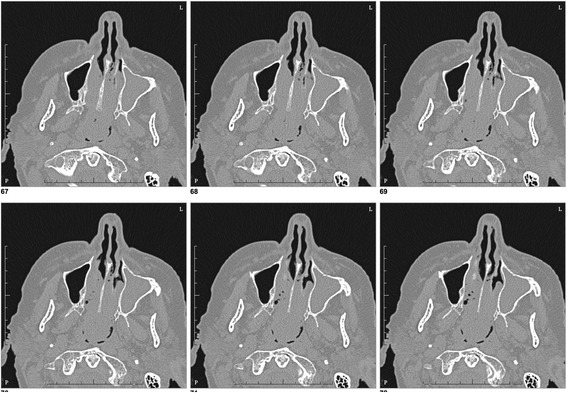
Computed tomography scan revealing a large soft tissue mass opacifying the left maxillary, ethmoidal, frontal, and sphenoid sinuses

On examination, her vital signs were within normal range and the rest of the physical and neurological examinations were normal. Her blood pressure was within normal range at 120/70 mmHg. A nasal speculum examination found a large tissue growth in her left nasal cavity and a subsequent computed tomography (CT) scan revealed a large soft tissue mass opacifying the left maxillary, ethmoidal, frontal, and sphenoid sinuses – there was no intracranial extension (Fig. 1). Laboratory findings including a full blood count, renal function, and liver function were unremarkable. She was referred to the Ear, Nose, and Throat surgical team and underwent a debulking surgery.

On frozen section the provisional diagnosis was a poorly differentiated squamous cell carcinoma on the basis of what was thought to be evidence of squamous differentiation in amongst a population of poorly differentiated cells. Only after processing and examination of the whole lesion was it appreciated that the tumor had both mature and immature components from more than one germ cell line.

**Fig. 2:**
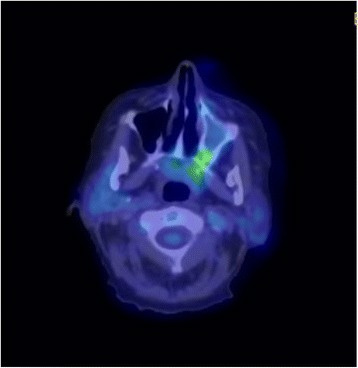
Positron emission tomography scan after debulking surgery showing ongoing soft tissue thickening in the left nasal cavity and the maxillary, ethmoid, and frontal sinuses

**Fig. 3:**
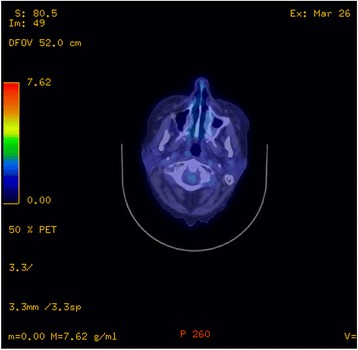
Positron emission tomography scan post-completion of radiotherapy showing no fluorodeoxyglucose-avid disease

A postoperative positron emission tomography (PET) scan revealed some ongoing soft tissue thickening in her left nasal cavity and her maxillary, ethmoid, and frontal sinuses with no evidence of distal fluorodeoxyglucose (FDG)-avid disease (Fig. 2). She received postoperative IMRT 60 Gy in 30 fractions to her sinuses. The treatment technique was a seven-field step-and-shoot IMRT plan with 80 control points. This was prescribed to a dose of 60 Gy. The difficulty in planning this was the close proximity of critical structures including brainstem and optic chiasm; the dose to the planning target volume (PTV) near these structures was reduced to 54 Gy in order to maintain tolerable dose constraints to these adjacent organs at risk. This resulted in a significant resolution of the tumor with no evidence of FDG-avid disease in her left maxillary sinus or nasal region on a repeat PET scan 6 months post-completion of radiotherapy (Fig. 3). We have followed her up closely at 3 monthly intervals and she had no evidence of local recurrence on nasal endoscopic examination at her 2-year follow-up.

## Discussion

SNTCS is a highly aggressive and rare malignant tumor with epithelial, mesenchymal, and neuroepithelial origins. The term was first used by Heffner and Hyams based on a case series with 20 patients with SNTCS [3]. This is a rare tumor with less than 100 cases being reported in the medical literature [5]. It is found mostly in adults with only a handful of pediatric cases being reported, and has a male predilection [6]. It is most commonly found in the nasal cavity, with involvement of the ethmoid sinus in approximately half of the patients and the maxillary sinus in approximately one-quarter of patients [7]. Intracranial extension is fairly common given the aggressive nature of the tumor [8].

**Fig. 4:**
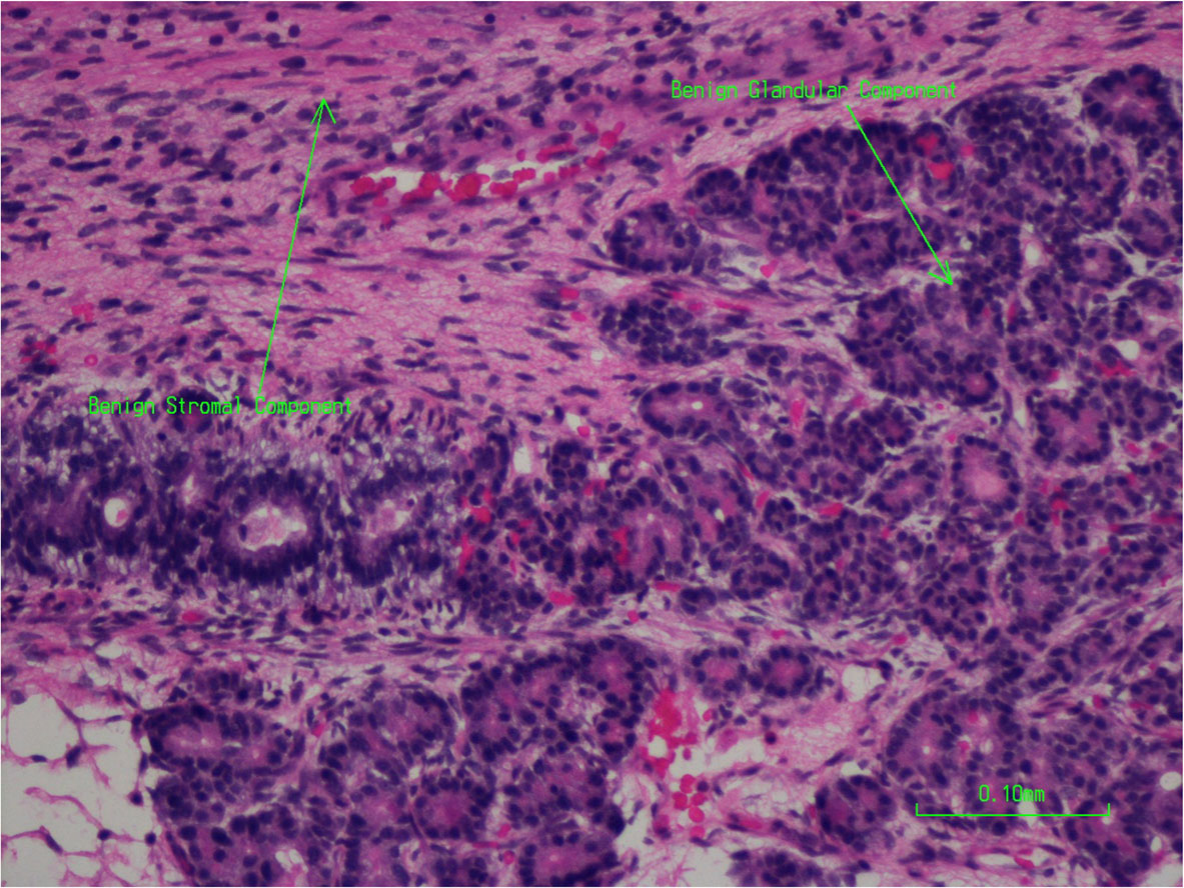
Mature benign glandular and stromal components of tumor

**Fig. 5:**
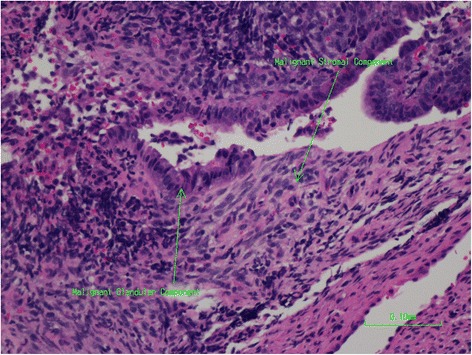
Immature malignant glandular and stromal components of tumor

Only after formal examination of the whole specimen was it recognized both histologically and on immunohistochemistry that the tumor was a heterogeneous mixture of components from all three germ cell layers at varying degrees of differentiation. These included both mature benign glandular and stromal components (Fig. 4) along with immature malignant glandular and stromal components (Fig. 5). Parts of the undifferentiated component showed either CD-99 or synaptophysin positivity. This combination of features is considered to give rise to the diagnosis of a sinonasal teratocarcinosarcoma (malignant teratoma). It can then be seen that inadequate sampling can lead to frequent misdiagnosis particularly due to the rare nature of the tumor [9].

On clinical examination, patients often present with nasal obstruction and epistaxis; however, they can also present with anosmia, headache, visual changes, and neurological symptoms [6].

The prognosis in SNTCS remains unclear, however, it is an aggressive tumor and recurrence and metastasis are common, with a recent systematic review of 71 cases quoting figures of approximately one-quarter having recurrence and 10% having metastasis [7]. Local invasion with intracranial extension can also occur. In Heffner and Hyams’ review of 20 cases, 12 died within 3 years with an average survival of 1.7 years [3]. However, in the eight cases that survived beyond 3 years there was no further recurrence or metastasis.

The optimal approach to treatment remains unclear in this condition due to the lack of large-scale studies given the rarity of the disease. A systematic review by Misra *et al*. consisting of 86 patients showed that the most common treatment modality was surgical resection followed by radiation therapy, with 51 cases out of 86 utilizing this approach [7]. The average dose of radiotherapy used was 55 Gy, and two case reports by Tokunaga *et al*. and Peng *et al*. have utilized IMRT with good effect, with no recurrence at 2 and 3.5 years respectively [10, 11]. Our case supports the use of IMRT given the excellent outcome in our patient with minimal side effects 2 years post-radiotherapy.

The role of adjuvant chemotherapy remains unclear; there is some suggestion that adjuvant chemotherapy can lead to better survival outcomes. Misra *et al*. reported an 88.8% survival rate at follow-up after an average of 32 months for those who received adjuvant chemotherapy in addition to surgery and radiation, as compared to 56.5% at 45 months for those who received surgery and radiation only [7, 12]. However, given the small number of cases that have utilized this approach a larger case series will lead to a better understanding of the efficacy of adjuvant chemotherapy for SNTCS.

## Conclusions

This case demonstrates that radiotherapy alone can be an effective and well-tolerated treatment modality in patients with SNTCS who have residual disease post-surgery. Further larger scale studies should be conducted to clarify if there is a role for adjuvant chemotherapy.

## Abbreviations

ADLs: Activities of daily living
CT: Computed tomography
ECOG: Eastern Cooperative Oncology Group
FDG: Fluorodeoxyglucose
IMRT: Intensity-modulated radiation therapy
PET: Positron emission tomography
PTV: Planning target volume
SNTCS: Sinonasal teratocarcinosarcoma
